# Maternal Feeding Controls Fetal Biological Clock

**DOI:** 10.1371/journal.pone.0002601

**Published:** 2008-07-02

**Authors:** Hidenobu Ohta, Shanhai Xu, Takahiro Moriya, Masayuki Iigo, Tatsuya Watanabe, Norimichi Nakahata, Hiroshi Chisaka, Takushi Hanita, Tadashi Matsuda, Toshihiro Ohura, Yoshitaka Kimura, Nobuo Yaegashi, Shigeru Tsuchiya, Hajime Tei, Kunihiro Okamura

**Affiliations:** 1 Center for Perinatal Medicine, Tohoku University Hospital, Sendai, Japan; 2 Department of Obstetrics and Gynecology, Tohoku University Hospital, Sendai, Japan; 3 Department of Pediatrics, Tohoku University Hospital, Sendai, Japan; 4 Department of Cellular Signaling, Graduate School of Pharmaceutical Sciences, Tohoku University, Sendai, Japan; 5 Department of Applied Biochemistry, Faculty of Agriculture, Utsunomiya University, Tochigi, Japan; 6 Tohoku University Biomedical Engineering Research Organization, Sendai, Japan; 7 Research Group of Chronogenomics, Mitsubishi Kagaku Institute of Life Sciences, Tokyo, Japan; University of Sydney, Australia

## Abstract

**Background:**

It is widely accepted that circadian physiological rhythms of the fetus are affected by oscillators in the maternal brain that are coupled to the environmental light-dark (LD) cycle.

**Methodology/Principal Findings:**

To study the link between fetal and maternal biological clocks, we investigated the effects of cycles of maternal food availability on the rhythms of *Per1* gene expression in the fetal suprachiasmatic nucleus (SCN) and liver using a transgenic rat model whose tissues express luciferase *in vitro*. Although the maternal SCN remained phase-locked to the LD cycle, maternal restricted feeding phase-advanced the fetal SCN and liver by 5 and 7 hours respectively within the 22-day pregnancy.

**Conclusions/Significance:**

Our results demonstrate that maternal feeding entrains the fetal SCN and liver independently of both the maternal SCN and the LD cycle. This indicates that maternal-feeding signals can be more influential for the fetal SCN and particular organ oscillators than hormonal signals controlled by the maternal SCN, suggesting the importance of a regular maternal feeding schedule for appropriate fetal molecular clockwork during pregnancy.

## Introduction

Most living organisms exhibit circadian rhythms, oscillations with a period of approximately 24 hours, in their behaviors and physiological functions, including activity, sleep, metabolism and body temperature. Circadian rhythms normally entrain to daily environmental cycles and free-run with a period of approximately 24 hours (called “circadian period”) in the absence of environmental cues [1]. Circadian period is remarkably precise for each species and differs slightly from 24 hours [2], [3]. In mammals, the circadian timing system is organized as a hierarchy of multiple organ oscillators [4], [5]. Among them the suprachiasmatic nuclei (SCN) of the anterior hypothalamus function as the master pacemaker at the top of the hierarchy, which coordinates clocks in peripheral organs such as the heart, lung, liver, kidney, pancreas and uterus [6]. At the molecular level, cellular clocks in the organs are controlled by autoregulatory transcriptional and translational feed back loops of key “clock genes”, in which BMAL1 and CLOCK proteins drive expression of the *Per* and *Cry* genes while the PER and CRY proteins in turn suppress the transcription of their own genes. In the circadian system, the light-dark (LD) cycle is the most reliable and effective external signal that synchronizes (entrains) biological rhythms with the environment. In mammals, photic information is perceived by specialized retinal photoreceptors and conveyed directly to the SCN of the hypothalamus, which is hypothesized to transfer circadian information to the other organs through hormonal signals or the nervous systems [1].

During fetal development, however, the situation is different. Fetuses do not respond directly to the entraining effects of light, but the timing of their biological clock is nevertheless coordinated with the environmental light-dark cycle. This prenatal entrainment of the fetal biological clock is the result of communication of time-of-day information from the mother to fetus in the uterus [7], [8]. In addition, based on postnatal behavioral rhythms, maternal-fetal communication of circadian phase is considered to be disrupted by destruction of the maternal SCN, suggesting that the fetal clock is regulated by the maternal entraining signal [7], [9]. Previous studies focused on the possibility that the maternal signal comes from hormones regulated by the maternal SCN since fetuses are anatomically separated from maternally-originated tissues by the placenta and maternal-fetal neural communication does not exist. Removal of selected maternal endocrine organs (pineal, pituitary, ovary, adrenal, thyroid and parathyroid), however, does not seem to disrupt maternal-fetal communication of circadian phase in the rat fetal clock, indicating that the rhythmic hormonal outputs from these glands may not be necessary [10].

In this study, we focused on a maternal signal, which is not directly controlled by the maternal SCN, by using a restricted feeding (RF) schedule to examine the possibility of feeding-related factors being a synchronizer for the fetal SCN. When food is available only for a limited time each day in an RF schedule, rats increase their locomotor activity 2 to 4 hours before the onset of food availability [11]. Entrainment of anticipatory locomotion by RF occurs independently of the LD cycle, suggesting that the circadian oscillators entrained by RF are distinct from those entrained by light. Surprisingly, RF does not influence the phase of clock gene expressions in the adult SCN but does influence locomotor activity and the clock gene rhythmicity in the other organs [12]–[14]. Thus, this is an ideal model to test the effect of maternal circadian signals independent of the maternal SCN on the fetuses. Moreover, synchronization can be found between the phase of locomotor activity of an SCN-lesioned mother rat who has been entrained through RF while still pregnant, and that of her newly born offspring, suggesting that fetal clocks can be controlled by RF on mothers [15]. With a transgenic rat model in which the mouse *Per1* gene promoter has been linked to a luciferase reporter, we continuously monitored the rhythmic expression of *Per1*, one of the key “clock genes”, by recording light emission from tissues *in vitro* [4]. We used this model to investigate the effects of maternal feeding on the communication between mother and the fetal SCN during pregnancy.

## Results and Discussion

**Figure 1:**
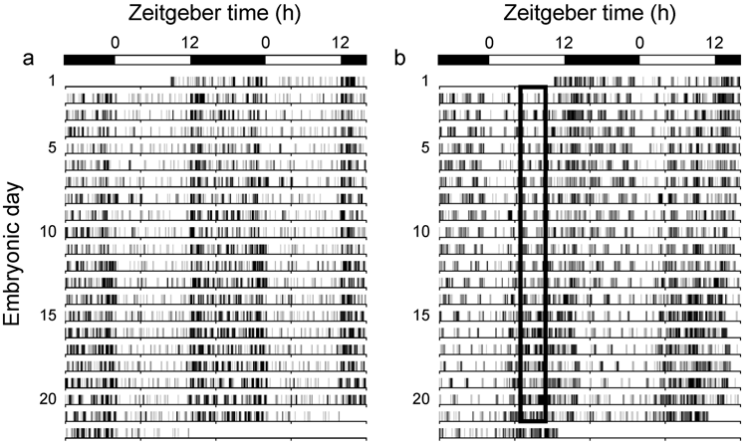
Representative locomotor activity records from pregnant *Per1-luc* transgenic rats. (a) shows activity in an animal under *ad lib* feeding. (b) shows activity in animals given access to food for 4 hours each day (the restricted feeding (RF) group). The open boxes in (b) indicate the daily food-access interval. The bars at the top indicate the light period in white and dark period in black. For rats in the RF group, food access was restricted to a 4-hour period at zeitgeber time (ZT) 5–9 for 21 days of pregnancy (where ZT0 is lights on and ZT12 is lights off). RF in (b) resulted in typical anticipatory activity occurring before food access. During the RF, activity is generally increased and the nighttime activity is shifted forward toward the food-access period of daytime.

**Figure 2:**
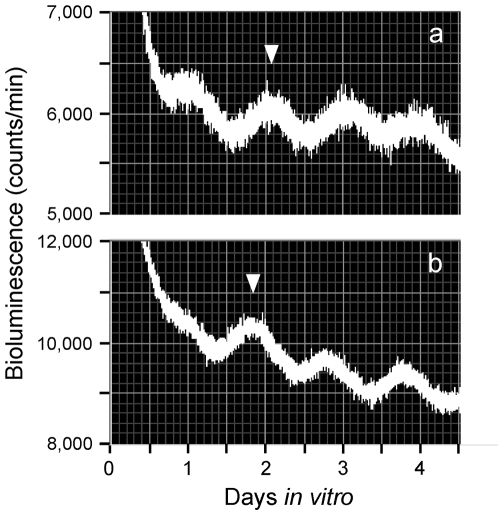
Rhythms of light emission by fetal SCN explants. Shown are raw data from (a) a fetus of an *ad lib* fed control pregnant animal and (b) a fetus of a pregnant animal that had been exposed to a 4-hour RF regimen for 21 days after mating. Because the pattern of light emission is quite variable during the first 12 to 14 hours after explantation, we consider that the phase of the tissue *in vivo* is best reflected by the phase of the peak during the first full subjective day (1 to 2.5 days after explant) as previously described [13]. The phase of these peaks is consistent from animal to animal (Fig. 4). Here, the phase statistically chosen is indicated by the inverted triangles.

**Figure 3:**
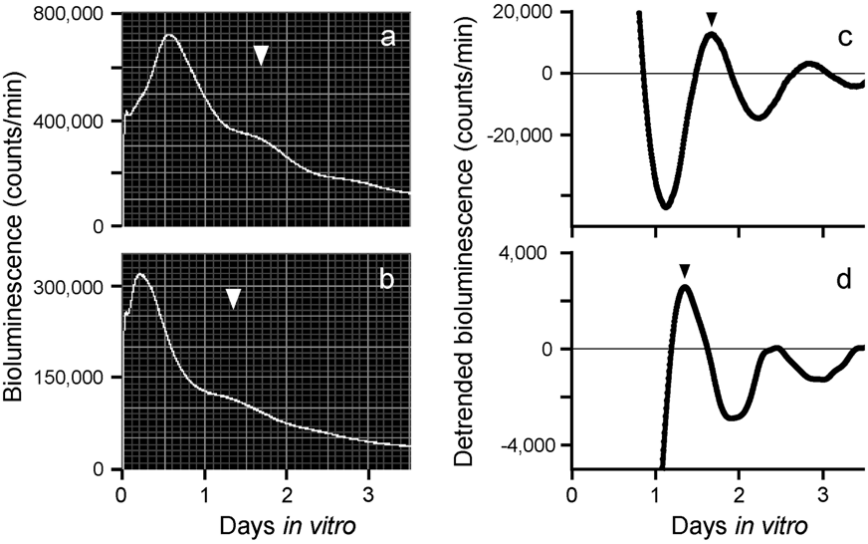
Rhythms of light emission by fetal liver explants. Shown are raw data (a) and detrended data (c) from a fetus of an *ad lib* fed control pregnant animal. (b) and (d) show raw and detrended data, respectively, from a fetus of a pregnant animal that had been exposed to a 4-hour RF regimen for 21 days after mating. The peak of the phase during the first full subjective day (1 to 2.5 days after explant) as statistically chosen is indicated by the white and black inverted triangles. The white inverted triangle in (a) and the black inverted triangle in (c) indicate the same peak time statistically chosen, as do the white and black triangles for (b) and (d), respectively.

**Figure 4:**
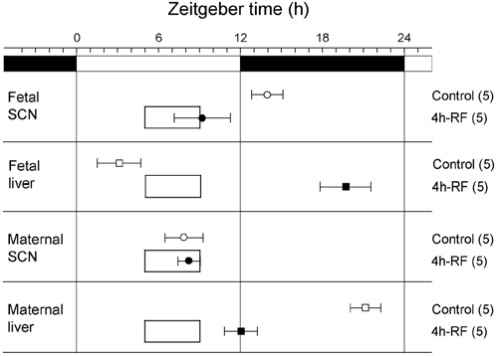
Effects of 4-hour restricted feeding on tissue luciferase rhythmicity. The average times (±s.d., shown by error bars) of peaks from the different tissues are plotted against the LD cycle shown at the top of each panel. The timing and duration of food availability at ZT5-9 is indicated by open boxes in each section. The sample size is shown in parentheses. The phases of fetal SCN, fetal liver and maternal liver rhythmicity were significantly different from control values in all groups of RF rats (one-way ANOVA, p<0.01); the phase of maternal SCN rhythmicity was not significantly different between control and 4h-RF groups.

We first exposed pregnant *Per1-luc* rats to an RF regimen, in which food was available only for 4 hours during the light portion of a 12-hour:12-hour LD cycle, and recorded their locomotor activity, for 22 days of pregnancy after mating. Within 10 days, the rats began to increase their behavioral activity a few hours before food became available, and also shifted their locomotor-active period from night to daytime (Fig. 1). After 21 days of RF and one following day of fasting, we sacrificed the animals; explanted both the fetal SCN and liver as well as the maternal SCN and liver and measured luciferase activities from each tissue *in vitro* (Fig. 2, Fig. 3 and Fig. 4). Despite the marked effects of this regimen on maternal locomotor behavior, the phase of the maternal SCN rhythm was unaffected (one-way ANOVA, p>0.05; the peak times for ad lib and restricted feeding were 43.9±1.4 h and 44.2±0.8 h (mean±s.d., throughout) respectively) and remained phase-locked to the light cycle, even after 21 days of RF (Fig. 4). This result is consistent with previous studies which report that RF does not entrain the adult SCN and supports the general notion that entrainment to cycles of food availability does not directly involve the maternal SCN [12]–[14]. In contrast, the circadian clock in the fetal SCN was entrained by the 4-hour RF regimen (Fig. 2, Fig. 4) and phase-advanced by 4.7 h (one-way ANOVA, p<0.01; the peak times for ad lib and restricted feeding were 50.0±1.1 h and 45.2±2.1 h respectively). This suggests that the fetal SCN may have a unique ability to adapt temporally to changes in the maternal feeding pattern unlike the adult SCN. The fetal SCN showed prominent *Per1-luc* circadian rhythms (23.3±1.7 h, n = 5, in the *ad lib* feeding and 22.5±2.0 h, n = 5, in the RF for the calculated free running periods of the fetal SCN *in vitro*; no statistical difference between the two groups) with smaller (0.32-fold) trough-to-peak amplitudes compared to those of the maternal SCN.

Our finding of *Per1-luc* circadian rhythms in the rat fetal SCN differs from the findings of some previous studies that did not detect clear *Per1* circadian rhythms in the rodent fetal SCN by *in situ* hybridization [16]–[21]. In addition to possible variation due to putative strain and species difference, this discrepancy might be explained by the technical difficulty in measuring the low-amplitude *Per1* circadian expressions in the developing SCN by *in situ* hybridization, which requires a delicate combination of the proper affinity of designed probes to the target *Per1* mRNA and the appropriate film exposure time for successful detection of weak radio-labeled *Per1* expression without over-saturation. To ensure correct measurements, we surgically made purely coronal SCN slices from *Per1-luc* transgenic fetal rats to detect *Per1-luc* signals directly from the fetal SCN and eliminate the background *Per1-luc* expressions from other neighboring brain tissue. In addition, highly-sensitive photo multiplier tubes were used to capture the low level *Per1-luc* signals. Our findings in the present study are consistent with previous reports on circadian rhythms in the firing rates and metabolic activities of the rat fetal SCN [7], [22], [23] and are also supported by *in-vivo* imaging data which demonstrated day-night differences in *Per1-luc* expressions throughout the whole body of *Per1-luc* rat fetuses during the late gestational period [24].

The circadian phases in the fetal liver in RF also showed phase-advance, advancing by 7.4 h compared to those in *ad lib* feeding (n = 5 for each group, one-way ANOVA, p<0.01; the peak times for ad lib and restricted feeding were 39.1±1.6 h and 31.7±1.8 h respectively; Fig. 3, Fig. 4), indicating that the fetal liver clock as well as the fetal SCN was entrained by the maternal RF regimen. The relatively smaller phase advance in the fetal SCN compared to the fetal liver in maternal RF may be explained by a possible competition in the fetal SCN between a stronger maternal-feeding based synchronizer and other unknown signals which subtract from the phase advance induced by maternal RF. Although *Per1-luc* circadian rhythms were detected in the fetal liver (23.5±1.1 h, n = 5, in the *ad lib* feeding and 24.2±1.5 h, n = 5, in the RF for the calculated free running periods; no statistical difference between the two groups), the oscillations were damped with smaller (0.28-fold) trough-to-peak amplitudes compared to those of the maternal liver. This is in contrast to fetal SCN circadian rhythmicity which displays more distinct oscillations, suggesting a more immature nature of the molecular clock in the fetal liver at this developmental stage. The phases in the maternal liver in RF also phase-advanced by 9.1 h compared to those in *ad lib* feeding as previously reported (n = 5 for each group, one-way ANOVA, p<0.01; the peak times for ad lib and restricted feeding were 57.1±1.1 h and 48.0±1.2 h, respectively; Fig. 4) [13] .

Our data invite a reexamination of the previous models of maternal-fetal communication in the mammalian circadian system. Previous studies have been trying to find the signals between maternal and fetal SCN based on a hypothesis that signals regulated by the maternal SCN, which is entrained by a daily light-dark cycle, exclusively control the fetal SCN [9], [10]. This study, however, suggests that maternal-feeding signals might be an alternative mechanism controlling the fetal SCN. Since RF did not affect the maternal SCN (Fig. 4), the present study indicates that fetal SCN are not directly controlled by hormonal signals regulated by maternal SCN but by maternal feeding. Further study is still required to identify possible alternative signals from mother to fetuses in the RF paradigm. A more direct way to confirm the findings of this study would be to employ more advanced *in-vivo Per1-luc* imaging of the fetal SCN in the pregnant uterus, however, such a technique has yet to be developed [25], [26].

Our results also have potential practical importance particularly for both normal and abnormal pregnancies [27]–[29]. During pregnancy, a regular daily-lifestyle schedule with appropriate amounts of sleep and nutrition is regularly recommended for pregnant women to achieve healthy fetal growth. So far studies on maternal feeding have only focused on the nutritional requirements for healthy fetal growth. However, this study illustrates that the maternal feeding schedule itself also has powerful effects on fetal physiology by influencing time information in maternal-fetal communication. In this rodent study, fetal biological clock as well as maternal behavior was strongly influenced by RF even though the maternal SCN remained adjusted to the light-dark schedule. This indicates that maternal-feeding signals can be more influential on fetuses than the maternal SCN during pregnancy and that perhaps time information relating to the LD cycle is relayed to the fetus via the mother's own LD-cycle based feeding cycle. The most appropriate maternal feeding schedules should be explored to achieve the sound physiology and healthy development of both fetuses *in utero* and preterm infants *ex utero*.

## Materials and Methods

### Animals and housing

Homozygous male and female transgenic *Per1-luc* rats (Japanese Wistar) expressing 6.7 kb of the mouse *Period1(Per1)* promoter driving firefly luciferase were used for this study. Timed-pregnant *Per1-luc* rats were housed individually in cages on a 12-hour:12-hour light-dark (LD) cycle (lights on at 08:00h and lights off at 20:00h; 200 lux at cage level during light period). The rats were exposed to a restricted feeding (RF) or *ad lib* feeding. For rats in the RF group, food access was restricted to a 4-h period at zeitgeber time (ZT) 5–9 for day 21 of pregnancy (where ZT0 is lights on and ZT12 is lights off). The RF rats adjusted the timing of their food intake to the limited feeding period within several days and also adjusted their daily food consumption to match normal daily intake levels as previously reported [11]. On day 22 of pregnancy, the last day of tissue culture, the pregnant rats under *ad lib* and restricted feeding were fasted to prevent the direct effects of food intake on the fetal SCN, to make sure not just one feeding event, but repeated feeding cycles over day 0–21 of pregnancy contributed to any changes in the fetal SCN rhythms. Locomotor activity of the rats was recorded by infrared motion sensors using an online system (Actograph System, Rapid Fire Computer, Japan). Animal care and use were reviewed and approved by the Committee for Animal Research of Tohoku University.

### Assessment of circadian periods and phases of the fetal and maternal SCN

Following behavioral assessment, both pregnant *Per1-luc* rats and their homozygous fetuses were sacrificed for recordings of SCN rhythmicity on day 22 of pregnancy, one day before expected birth. We sacrificed one fetus each from five different litters of both *ad-lib* and restricted-feeding type pregnant rats and their mothers at ZT11 and rapidly removed fetal and maternal brains (n = 5 for each) as well as maternal liver (n = 5). We also prepared a separate set of pregnant *Per1-luc* rats in *ad lib* and RF to sample the fetal livers (n = 5 for each group) in the same manner. The paired SCN (coronal sections of 300 µm thickness, made with a vibroslicer) and the liver tissues (1-mm thickness) were cultured on membrane inserts (Millicell-CM, Millipore, Bedford, MA) in 1 mL of medium (Dulbecco's modified Eagle's medium, Sigma, St.Louis, MO) supplemented with 10 mM HEPES (Sigma), 2% B27, 25 U/mL penicillin, 25 µg/mL streptomycin, 2.2 mg/mL NaHCO3, 4 mM L-glutamine, and 0.1 mM beetle luciferin (Promega, Madison, WI). Unless noted, medium ingredients were purchased from Invitrogen (Carlsbad, CA). Each culture was sealed in a Petri dish and maintained at 36°C in darkness. Bioluminescence was collected in counts per minute for 4.5 days without a medium change using a photomultiplier tube (HC8259MOD, Hamamatsu Corp., Shizuoka, Japan).

Phase, period, and amplitude were determined using modified published methods [30]–[32]. First, original data (1-min bins) were smoothed by an adjusting-averaging method with 2-hour running means as described [30]. Then the data set were detrended by subtracting the 24 hour running average from the raw data using Exmax software (gift from Mr. Tuyoshi Yaita and Dr. Shigenobu Shibata, Waseda University, Tokyo, Japan). Peak time was defined as the highest point in detrended data. The period of *Per1-luc* activity (recorded from 24 to 108 h *in vitro*) was assessed for each SCN culture and calculated by averaging the period between the first and second peaks and the period between the second and third peaks. The *Per1-luc* amplitude for an SCN culture was calculated as the difference between the first trough and second peak of the detrended curves of the bioluminescence. Statistical comparisons for these data from the RF and *ad lib* groups were performed by one-way ANOVA (p<0.05).
